# Molecular Epidemiology of Eastern Equine Encephalitis Virus, New York

**DOI:** 10.3201/eid1403.070816

**Published:** 2008-03

**Authors:** David S. Young, Laura D. Kramer, Joseph G. Maffei, Robert J. Dusek, P. Bryon Backenson, Christopher N. Mores, Kristen A. Bernard, Gregory D. Ebel

**Affiliations:** * New York State Department of Health, Albany, New York, USA; † School of Public Health, Albany, New York, USA; ‡ National Wildlife Health Center, Madison, Wisconsin, USA; § University of Florida, Vero Beach, Florida, USA; ¶ University of New Mexico School of Medicine, Albuquerque, New Mexico, USA

**Keywords:** Eastern equine encephalitis virus, Alphavirus, Togaviridae, Arboviruses, epidemiology, molecular, research

## Abstract

Perpetuation, overwintering, and extinction of eastern equine encephalitis virus (EEEV) in northern foci are poorly understood. We therefore sought to describe the molecular epidemiology of EEEV in New York State during current and past epizootics. To determine whether EEEV overwinters, is periodically reintroduced, or both, we sequenced the E2 and partial NSP3 coding regions of 42 EEEV isolates from New York State and the Eastern Seaboard of the United States. Our phylogenetic analyses indicated that derived subclades tended to contain southern strains that had been isolated before genetically similar northern strains, suggesting southern to northern migration of EEEV along the Eastern Seaboard. Strong clustering among strains isolated during epizootics in New York from 2003–2005, as well as from 1974–1975, demonstrates that EEEV has overwintered in this focus. This study provides molecular evidence for the introduction of southern EEEV strains to New York, followed by local amplification, perpetuation, and overwintering.

## Abstract 2

Southern strains are undergoing amplification, perpetuation, and overwintering in New York.

*Eastern equine encephalitis virus* (EEEV; genus *Alphavirus*: family *Togaviridae*) is maintained in an enzootic cycle between ornithophilic mosquitoes and birds. The virus causes disease in some avian hosts and in incidental hosts, such as horses and humans; case-fatality rate in humans is ≈33% (*1*). Virus activity has been detected in North and South America. In the United States, EEEV has been detected along the Gulf of Mexico and the Atlantic Seaboard as well as in inland foci near the Great Lakes, including upstate New York. The EEEV virion contains a single-stranded, positive-sense RNA genome of ≈12 kb. The 5′ end of the genome encodes 4 nonstructural proteins: NSP1, NSP2, NSP3, and NSP4. The structural proteins are encoded in the 3′ third of the genome and are translated from a subgenomic RNA, 26S, resulting in 5 protein products: C, 6K, E1, E2, and E3 (*2*). Previous sequencing studies analyzed genetic relationships of EEEV strains in the Western Hemisphere and compared strains distributed across widespread geographic regions (*3*–*6*). EEEV has 4 distinct genetic lineages; lineage I consists of highly conserved strains from North America, and lineages II–IV encompass strains from Central and South America (*3*).

Outbreaks of EEEV in New York have been observed periodically since 1952, when the virus was first detected in pheasants (*7*). Disease in humans and/or horses has been noted on Long Island, in the lower Hudson Valley, and in central upstate New York; the last known human case in New York occurred in 1983 in Onondaga County (*8*). Most EEEV activity in New York has occurred in counties bordering Oneida Lake in central upstate New York (Figure 1). Most of the activity in this region has been concentrated in the Big Bay–Toad Harbor Swamp complex in Oswego County and Cicero Swamp in Onondaga County (*8*). *Culiseta melanura* (Coquillett), the main enzootic vector of EEEV, breeds abundantly in these swamps (*9*). Localized epizootics in the counties of Oswego and Onondaga have been documented in a transmission focus during 1971–1977, 1982–1983, and 1990–1991 (*8*,*10*–*13*) and from 2003 to the present (2007; D.S. Young et al., unpub. data). Between these epizootic periods, EEEV was undetectable in horses and birds and only infrequently detected in mosquito pools (D.S. Young et al., unpub. data) (*8*). From 1992 through 1997 in upstate New York, EEEV was detected in 18 mosquito pools from Onondaga County (1994) and 3 mosquito pools from Oswego County (1996); no equine or avian cases were detected (D.S. Young et al., unpub. data). From 1998 through 2002, EEEV was not detected in mosquitoes or vertebrates in New York. However, in 2003, EEEV activity increased across New York with the emergence of the current epizootic (2003–2007) in the Onondaga and Oswego Counties region.

**Figure 1:**
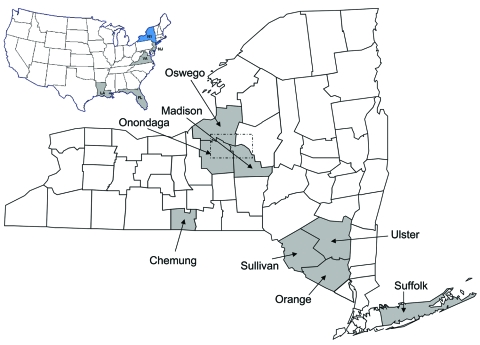
A) United States map showing locations of eastern equine encephalitis virus strains sequenced in this study. New York State (NY) highlighted in blue; New Jersey (NJ), Virginia (VA), Florida (FL), Louisiana (LA) highlighted in gray. Map courtesy of www.theodora.com/maps, used with permission. B) New York counties where eastern equine encephalitis virus (EEEV) strains have been located (shaded). Dotted box indicates focus of most EEEV activity.

Patterns of localized perpetuation, overwintering, and extinction of EEEV in transmission foci are poorly understood. To determine whether EEEV overwinters locally in temperate regions such as upstate New York or whether annual reintroduction is required to reinitiate the transmission cycle, we compared nucleotide sequences comprising the entire E2 coding region and part of the NSP3 coding region. We examined 35 strains isolated in New York during 1971–1975 and 2003–2005 and 7 strains collected along the Eastern Seaboard of the United States during 2002–2003. Using these data, we described the molecular epidemiology of EEEV strains collected during the current and past epizootics in New York.

## Materials and Methods

### EEEV Detection and Isolation

Isolates from New York State and the Eastern Seaboard were sequenced for this study (online Appendix Table, available from http://www.cdc.gov/EID/content/14/3/454-appT.htm). Strains originating outside of New York were isolated from avian serum samples, which were collected during a study conducted by the US Geological Survey and stored at –80°C until inoculation onto cell culture. All EEEV strains from within New York were collected from mosquito, avian, and equine samples that were submitted to the Wadsworth Center’s Arbovirus Laboratories as a part of surveillance efforts by the New York State Department of Health. EEEV strains isolated during 1971–1975 were obtained from our archives.

To obtain mosquito-derived EEEV strains, mosquitoes were collected from May through October by local county health department staff, who used standard miniature light or gravid traps. Mosquitoes were identified to the species level, and pooled samples of 10–50 mosquitoes in 2-mL microfuge tubes were submitted to the Arbovirus Laboratories for analysis. Tubes contained a steel ball-bearing (Daisy Brand, Rogers, AR, USA), and to each tube we added 1 mL of mosquito diluent (20% heat-inactivated fetal bovine serum [FBS] in Dulbecco’s phosphate-buffered saline with 50 μg/mL penicillin/streptomycin, 50 μg/mL gentamicin, and 2.5 μg/mL amphotericin B). Pools were homogenized by using a mixer mill (Retsch, Haan, Germany) at 24 cycles/s for 30 s and centrifuged for 4 min at 6,000 rpm at room temperature. The clarified homogenate was transferred to a new microcentrifuge tube and stored at –80°C until testing.

To obtain vertebrate-derived EEEV strains, samples of horse brains were submitted by the Wadsworth Center’s Rabies Laboratory, and samples of avian kidney, heart, and brain were submitted by the New York State Department of Environmental Conservation Wildlife Pathology Unit. Each horse brain was excised into 3 separate 1–3 mm^3^ sections, which were pooled for testing for each horse. Avian tissues were tested by excising the same-size portion from 1 of the tissues or by pooling sections of all 3 tissues. Excised tissues were placed in 2-mL microfuge tubes containing a ball-bearing and 1 mL of BA-1 virus diluent (M199 with Hanks’ salts and L-glutamine; [Mediatech, Herndon, VA, USA] in sterile distilled water with 0.05 M Tris(hydroxymethyl)aminomethane,1% bovine serum albumin, 0.35 g/L sodium bicarbonate, 100 U/mL penicillin, 100 U/mL streptomycin, 1 µg/mL amphotericin B, and 20% FBS). Hydrogen chloride was added to the diluent to bring the pH to 7.4. Samples were homogenized on the mixer mill at 24 cycles/s for 4 min and centrifuged for 5–8 min at 10,000 rpm at 4°C.

Virus was isolated by inoculating 100 mL of supernatant from mosquito pools, vertebrate tissues, or avian serum onto confluent monolayers of African green monkey kidney (Vero) cells grown in 6-well plates. Plates were incubated for 1 h at 37°C in 5% carbon dioxide, with gentle rocking every 15 min. To each well, 3 mL of maintenance medium (1× minimum essential medium with Earle’s salts [Invitrogen, Carlsbad, CA, USA], 2% heat-inactivated FBS, 1% 100× L-glutamine, 0.15% sodium bicarbonate, 1% penicillin–streptomycin, 0.1% amphotericin B, and 0.1% gentamicin diluted in sterile distilled water) was added, and plates were returned to the incubator and observed daily. If cytopathic effect was observed, the infecting virus was identified by either immunofluorescence assay (*14*) or reverse transcription PCR (RT-PCR) by using One-Step RT-PCR kit and protocol (QIAGEN, Valencia, CA, USA) as directed by the manufacturer. Primer sequences and cycling parameters are described elsewhere (*15*). Target bands were examined under UV light after electrophoresis on a 1.5% agarose gel. Samples that were positive for EEEV were stored at –80°C until use.

### RNA Extraction and RT-PCR

RNA was extracted from the Vero cell culture supernatant after a single passage, the original sample (no passage), or isolates with an unknown passage history by using the RNeasy kit (QIAGEN) as directed by the manufacturer. The entire E2 coding region was amplified in 2 separate reactions. To produce overlapping fragments, primers EEE8460 (5′-AGAATCCACACGAAACACTCACCA-3′) and EEE9200c (5′-ATCCGTGCAGGTGGTTGTATGGTC-3′) were used for the first reaction, and primers EEE9105 (5′-TCCACAGTGCCAAGGTGAAAA-3′) and EEE9887c (5′-CTGCAAGTGGGATAAGCGTCTG-3′) were used for the second reaction. The partial NSP3 coding region was amplified by using primers EEE4836 (5′-CAGAGCGAGTTTACAGATTACG-3′) and EEE5477c (5′-AACGGCGAACGACTGAA-3′). Sample RNA (5 μL) was added to 45 μL of One-Step RT-PCR master mix (QIAGEN) prepared according to the manufacturer. Several drops of mineral oil were added on top of each reaction. Samples were reverse transcribed for 30 min at 55°C and heat inactivated at 95°C for 5 min. To eliminate residual RNA, RNase was added to each reaction after reverse transcription. Samples were amplified by PCR according to the following thermocycler conditions: 94°C for 10 min; 39 cycles of 94°C for 30 s, 55°C for 30 s, 72°C for 45 s; and 72°C for an additional 10 min. PCR product (40 μL) was added to 4 µL of BlueJuice loading dye (Invitrogen, Carlsbad, CA, USA), and loaded onto an agarose gel containing 0.4 μg/mL of ethidium bromide. DNA underwent electrophoresis and was examined under UV light. Samples were sequenced on either an ABI 3100 or ABI 3700 automated DNA sequencer (Applied Biosystems, Foster City, CA, USA) at the Wadsworth Center Molecular Genetics Core facility. (Sequencing primers are available from the authors upon request.)

### Phylogenetic Analysis

Trace files were compiled by using the SeqMan module of Lasergene (DNAstar, Madison, WI, USA), with a minimum of 2-fold base-call redundancy required for all sequences. Consensus sequences for each strain sequenced in this study and reference strains obtained from GenBank were aligned by the ClustalV method (*16*) in the MegAlign module of Lasergene. Two phylogenetic trees were produced for the E2 coding region analysis: the main E2 tree, which included all strains included in this study, and the E2 subset tree, which contained only strains sequenced in this study and only those sequences from GenBank for which both E2 and NSP3 sequence data were available. The main E2 tree was generated by maximum likelihood in PAUP version 4.0b10 (Sinauer Associates, Sunderland, MA, USA) by using the HKY85+G model with relevant parameters estimated from the data. The robustness of the branching pattern was estimated by performing 1,000 neighbor-joining bootstrap replicates under the maximum-likelihood substitution model, also using PAUP; these values are presented on the maximum likelihood tree. The E2 subset and NSP3 trees were generated by neighbor-joining analysis with 1,000 bootstrap replicates by using the Kimura 2-parameter model in MEGA2 (*17*). Nucleotide sequences of newly sequenced strains were deposited in GenBank (see online Appendix Table for accession numbers). Nucleotide diversity (π) and the Tajima D statistics were computed by using DnaSP (*18*).

## Results

### Virus Strains

We sequenced 42 EEEV strains, which represented various geographical locations, hosts, and isolation dates (online Appendix Table). Of the 42 strains, 29 were from central upstate New York. Of those 29 strains, 13 were isolated during the 1970s epizootic, and the remainder were isolated during the current epizootic. The remaining strains sequenced were from various locations in the southeastern United States (Louisiana, Florida, and Virginia), New Jersey, and New York outside the central upstate focus (counties of Suffolk, Orange, Sullivan, Ulster, and Chemung) (Figure 1).

### E2 Phylogenetic Analysis

**Figure 2:**
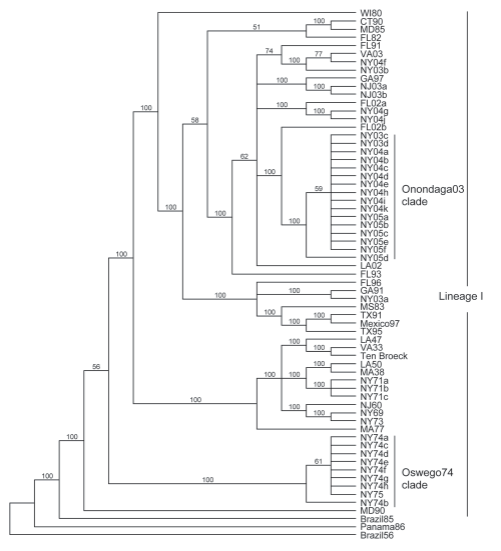
Maximum-likelihood phylogenetic tree of eastern equine encephalitis virus strains, based on the complete E2 coding sequence. Numbers at the nodes indicate bootstrap confidence estimated by 1,000 neighbor-joining replicates on the maximum-likelihood tree. The tree was rooted with lineage II (Brazil56), III (Panama86), and IV (Brazil85) strains.

**Figure 3:**
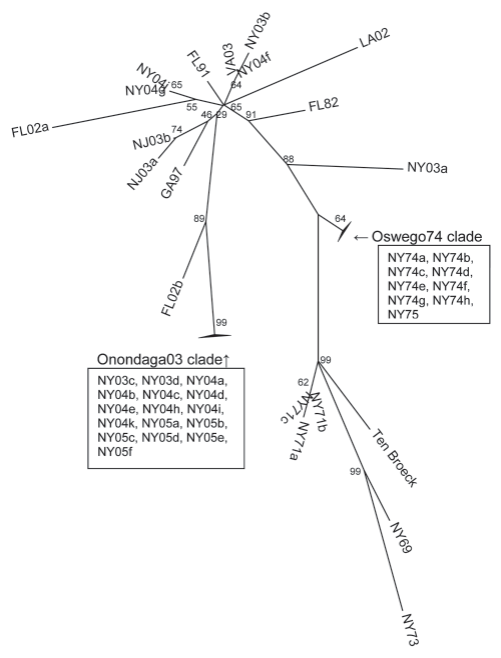
Phylogenetic tree of subset of lineage I eastern equine encephalitis virus strains, unrooted neighbor-joining analysis of E2 coding region. Strains included are identical to those used in the NSP3 coding region analysis.

**Figure 4:**
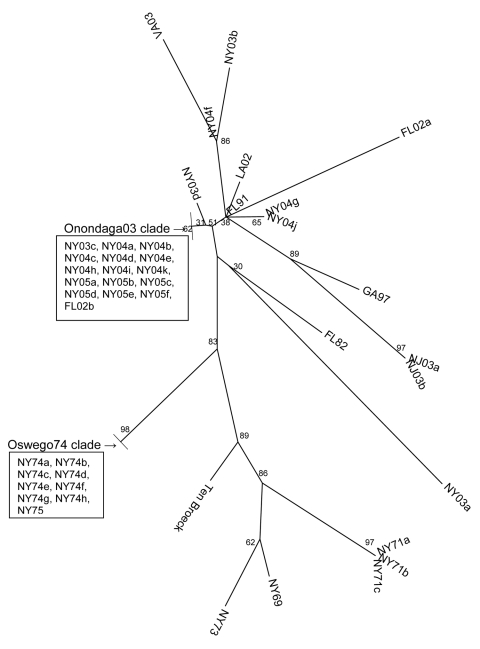
Phylogenetic tree of NSP3 coding region of subset of lineage I eastern equine encephalitis virus strains, unrooted neighbor-joining analysis.

Phylogenetic analysis of the E2 coding region demonstrated that all isolates sequenced in this study belonged to lineage I (Figure 2), and showed strong spatiotemporal clustering. The 3 strains isolated from Oswego County in 1971 (NY71a, NY71b, and NY71c) clustered together as a result of almost identical E2 coding regions. All 8 strains isolated from Oswego County in 1974 (NY74a–h) and the only strain isolated in 1975 (NY75) grouped together strongly and formed the Oswego74 clade (Figures 2–4). Strong clustering was also evident among strains isolated mainly from Onondaga County during 2003–2005 (the Onondaga03 clade, Figures 2–4). Of the 16 strains in this clade, 13 had identical E2 coding regions (data not shown). Both NY04g and NY04j grouped together and were isolated in close geographic proximity in Sullivan and Ulster Counties, respectively, in the lower Hudson Valley.

### Sequence Diversity and Phylogenetics

Examination of π, a measure of sequence diversity, confirmed the close relationships of sequences sampled during spatiotemporally defined epizootics. The π values for the Oswego74 and Onondaga03 clades were 0.00035 and 0.00030, respectively. The π value for the entire set of US strains included in our analysis was 0.01058, ≈30× greater than intraepizootic values. The Tajima test failed to reject neutrality in either the Oswego74 or Onondaga03 clade because of extremely low genetic diversity: only a few mutations were present in each grouping. Thus, the EEEV collected during New York epizootics is generally characterized by a high degree of sequence conservation with little genetic variation among spatially and temporally related strains.

However, spatiotemporal conservation was not absolute. NY73, isolated from a horse in Onondaga County in 1973 (NY73) was most genetically similar to NY69, isolated from a pheasant in Suffolk County on Long Island in 1969. Similarly, a strain isolated from a horse in Chemung County, New York (NY04k), fell into the Onondaga03 clade, which further demonstrated occasional relaxation of the otherwise strong time-space clustering of the strains studied. Consideration of strains from outside of New York provided additional instances in which the pattern of spatiotemporal clustering was broken. Well-supported subclades frequently contained southern progenitor strains that had been isolated years before they appeared in New York or New Jersey (Figures 2–4). Examples of this trend include the following: VA03 linked with NY03b and NY04f, GA97 linked with NJ03a and NJ03b, and FL02a linked with NY04g and NY04j. In addition, strain FL02b, isolated from an ovenbird (*Seiurus aurocapillus*) collected in Florida during 2002 was highly similar to the Onondaga03 clade.

To evaluate the possibility that analysis of different coding sequences would yield different results, we studied the NSP3 coding sequences of all strains for which sequence data were available or further sequencing was possible. GenBank sequence data for the NSP3 coding region was limited; therefore, another phylogenetic tree for the E2 coding region was produced by using a subset of lineage I strains for which the NSP3 sequences were available (Figure 3). This E2 subset tree was used for comparisons with the NSP3 tree to determine whether the trees shared similar topology. Phylogenetic analysis of the NSP3 coding region produced similar overall topologies (Figure 4); both trees recognized the major clades (Oswego74 and Onondaga03) and most of the minor subclades.

## Discussion

Samples that were newly sequenced for this study were selected to include relevant arthropod and vertebrate hosts (mosquitoes, birds, and horses) and were drawn from archives and ongoing arbovirus surveillance efforts. Additional sequences used in the analyses were obtained from GenBank. Strains collected from the current (2003–2005) epizootic were sequenced from primary field-derived material when possible. This was done to minimize the likelihood that adaptation to tissue culture, which has been observed for other alphaviruses (*19*), could bias our results. Because our analyses did not show strong passage-history–dependent clustering, it seems likely that the sequences from strains passed once in Vero cells are accurate representations of wild-type sequences. Further, strong sequence conservation among strains collected from taxonomically diverse hosts suggests that the source of the virus (mosquitoes or horses) was unlikely to result in sequence changes that might bias our conclusions.

Previous studies provided preliminary evidence that EEEV overwinters in upstate New York (*5*,*20*). The strongest such evidence was derived from RNA sequences and fingerprints that showed strong clustering of 11 strains collected in 1990 and 1991 (*5*). It has been suggested that EEEV may have been relatively isolated in upstate New York for up to several years and that the virus may have overwintered from 1990–1991 (*5*,*20*). The studies we present here document genetic conservation of strains throughout 3 transmission seasons and over 2 winters, which supports the observation that EEEV may overwinter in a relatively isolated upstate New York focus. Despite the accumulating molecular epidemiologic evidence for EEEV overwintering, the precise mechanisms are poorly understood. One potential overwintering mechanism is latent or chronic infection of wild birds. In such a scenario, springtime viral recrudescence might reinitiate the transmission cycle each new season (*21*,*22*). However, the results of a serologic survey of wild birds in upstate New York during 1986–1990 failed to support this notion, showing no consistent evidence for the current or recent infection of after-hatch year birds with EEEV early in the transmission season (*23*). Transovarial transmission in mosquitoes also has been hypothesized as a means for overwintering, but it has not been convincingly demonstrated naturally or experimentally (*24*–*26*). The main epizootic vector, *Cs. melanura*, overwinters in the larval stage (*27*), so the virus would need to perpetuate in these larvae. As an alternative, predatory birds could acquire the virus by feeding on infected prey, perhaps enabling the virus to persist through winter without mosquitoes. A previous study describing the isolation of *West Nile virus* (WNV; *Flaviviridae: Flavivirus*) from a hawk in New York in winter (*28*) supports this idea. However, conclusive evidence for this theory does not yet exist. Moreover, despite several ecologic studies of EEEV in upstate New York, the mode of long-term persistence in enzootic transmission cycles remains obscure. Accordingly, the molecular epidemiologic studies described here were undertaken to determine more convincingly whether EEEV overwinters locally in upstate New York.

Sequence data from the E2 and NSP3 coding regions of EEEV strains collected during 2 independent multiyear epizootics, 1 in the 1970s and 1 in the 2000s, enabled us to use a molecular approach to examine whether EEEV overwinters in temperate regions. We observed strong spatiotemporal clustering of EEEV strains, including several strains that were identical in their E2 coding region, collected in a single focus over the course of several years. For example, 16 EEEV strains isolated during 2003–2005 form the Onondaga03 clade, which strongly suggests that the virus overwintered there. The probability that this highly conserved genotype was reintroduced in each of 3 consecutive years seems quite low. The Oswego74 clade also supports overwintering of EEEV. Collectively, these data indicate that EEEV was perpetuated locally through several winters in upstate New York during elevated epizootic activity periods, with 1 dominant genotype circulating in the focus.

The history of EEEV activity in New York suggests that transmission dynamics are not uniform and that periods of relative intensity punctuate interepizootic periods, when virus is undetectable or detectable only infrequently in mosquito pools (D.S. Young et al., unpub. data) (*8*,*29*). Phylogenetic analyses suggest that epizootics occur after reintroduction of novel EEEV genotypes from southern progenitor strains. For example, the Onondaga03 clade groups strongly with the strain FL02b, which was isolated from an ovenbird in Florida in 2002. The ovenbird resides in Florida and Central America in the winter and migrates north to Canada and the northern United States, including upstate New York, in the summer (*30*). The current epizootic therefore appears to be the result of introduction of a southern EEEV strain similar to FL02b in 2003. Although the results presented here cannot determine the precise mode of EEEV transport along the Eastern Seaboard (e.g., trade winds have also been suggested as a mechanism for moving infected mosquitoes (*31**,**32*), it seems likely that migratory birds are involved in virus trafficking to at least some degree.

The E2 and NSP3 phylogenetic trees demonstrate that some subclades contain southern strains isolated years before genetically similar northern strains. Such is the case with subclades GA97, NJ03a, NJ03b and VA03, NY03b, NY04f and in the E2 trees only (FL02a, NY04g, NY04j). This pattern provides evidence for regular reintroduction of EEEV into enzootic areas of New York State and New Jersey. The subclades VA03, NY03b, NY04f suggest that northward migrating birds brought the virus from the South into New York in 2003. However, the low number of samples from the Eastern Seaboard limits definitive conclusions. For this study we included only 7 samples isolated from the Eastern Seaboard in 2002 and 2003. Sequencing additional southern strains isolated in 2004 or later, to characterize the genetic relationships between northern and southern strains in greater depth, would be beneficial.

A defining feature of the collection of EEEV sequences analyzed here is the genetic conservation within and between epizootics. We observed values of genetic diversity in the Onondaga03 and Oswego74 clades that were surprisingly similar and very low (0.00030 and 0.00035, respectively). These values are ≈10-fold lower than π observed in a sample of WNV sequences collected during 1999–2003 in Suffolk County, NY (0.00241) (*33*). The causes for the strikingly different patterns of genetic diversity observed in these 2 viral systems could include 1) increased movement of WNV-infected birds or mosquitoes compared with EEEV-infected birds or mosquitoes, leading to more frequent introduction of novel genotypes, 2) a higher replicase error rate, and 3) relaxed selective constraint in hosts or vectors of WNV relative to EEEV. Regardless of cause, the basic evolutionary dynamics of these RNA viruses appear to differ markedly.

Overall, our data support the previous findings of Weaver et al. (*5*,*20*) and provide new insights into the ecologic and evolutionary dynamics of an ongoing EEEV epizootic. We provide evidence that the virus is introduced from southern progenitor strains which, if they become established, overwinter in upstate New York; few new genotypes were successfully introduced into the epizootic focus. Our results also highlight the relative spatiotemporal genetic conservation of the virus. To facilitate a more detailed understanding of patterns of perpetuation and spread of this important zoonotic pathogen, future monitoring of EEEV activity should focus on sampling along the entire Eastern Seaboard.
